# BMI Increases in Individuals with COVID-19-Associated Olfactory Dysfunction

**DOI:** 10.3390/nu15214538

**Published:** 2023-10-26

**Authors:** Brandon J. Vilarello, Patricia T. Jacobson, Jeremy P. Tervo, Liam W. Gallagher, Francesco F. Caruana, Joseph B. Gary, Tiana M. Saak, David A. Gudis, Paule V. Joseph, Terry E. Goldberg, D.P. Devanand, Jonathan B. Overdevest

**Affiliations:** 1 Vagelos College of Physicians and Surgeons, Columbia University, New York, NY 10032, USA; 2 Department of Otolaryngology-Head and Neck Surgery, New York-Presbyterian/Columbia University Irving Medical Center, New York, NY 10032, USA; 3 National Institute of Alcohol Abuse and Alcoholism, Section of Sensory Science and Metabolism & National Institute of Nursing Research, Bethesda, MD 20892, USA; 4 Department of Psychiatry, New York-Presbyterian/Columbia University Irving Medical Center, New York, NY 10032, USA

**Keywords:** BMI, olfaction, chemosensation, COVID-19, nutrition

## Abstract

(1) Background: Reports suggest COVID-19-associated olfactory dysfunction (OD) may result in alterations in dietary behaviors and perceived weight change, but few studies using psychophysical evaluation of post-COVID-19-associated chemosensory dysfunction and body mass index (BMI) exist. The purpose of this study is to assess the impact of both quantitative and qualitative features of COVID-19-associated OD on BMI; (2) Methods: Recruitment of thirty-one participants with self-reported OD in the form of quantitative loss with and without qualitative features. Surveys with questions specific to qualitative olfactory function, Sniffin’ Sticks tests, and BMI measures were completed at two visits, one year apart. Group differences were assessed with Wilcoxon signed-rank tests and the Holm–Bonferroni method; (3) Results: Individuals with persistent quantitative OD (*n* = 15) and self-reported parosmia (*n* = 19) showed statistically significant increases in BMI after 1 year (*p* = 0.004, adjusted *α* = 0.0125; *p* = 0.011, adjusted *α* = 0.0167). Controls with transient quantitative OD (*n* = 16) and participants without self-reported parosmia (*n* = 12) showed no statistically significant changes in BMI over the same time period (*p* = 0.079, adjusted *α* = 0.05; *p* = 0.028, adjusted *α* = 0.025); (4) Conclusions: This study shows an association between COVID-19-associated OD and BMI, suggesting olfaction may play a role in altering dietary habits and nutrition in this population. Larger study cohorts are needed to further evaluate this relationship.

## 1. Introduction

The COVID-19 pandemic has affected many aspects of nutrition, both directly through acute illness and indirectly through societal changes. Several factors impact dietary and eating behaviors, such as knowledge of healthy/unhealthy foods, cultural preferences, socioeconomic factors, availability of certain foods, marketing of foods, psychological factors, social elements (e.g., interpersonal contacts), and chemosensory percepts (gustation and olfaction) [1].

A well-known sequela of COVID-19 is olfactory dysfunction (OD), or changes in how individuals perceive smells [2,3]. While most studies focus on quantitative smell dysfunction or reduced magnitude of the sense of smell, which includes hyposmia and anosmia, COVID-19 also causes qualitative olfactory distortions, known as parosmia [4,5]. Parosmia manifests as the perception of odors as being different than they should smell, most commonly where pleasant odors are perceived as foul. Chemosensory dysfunction, which also includes perception of taste, can significantly affect nutrition and eating habits, and normal olfaction and gustation are important for maintaining a normal metabolism [6]. Individuals may experience reduced appetite, altered food preferences, and a decreased enjoyment of food, which can potentially lead to inadequate nutrient intake, malnutrition, and overnutrition [7]. A mouse experiment examining OD and BMI found that mice with reduced olfactory input exhibit stronger sympathetic responses leading to lipolysis and weight loss [8]. On the other hand, poorer olfactory function has previously been shown to correlate with obesity, suggesting that reduced olfactory function may delay satiety and lead to excessive weight gain [9]. Limited studies looking specifically at the association between hyposmia/anosmia and body mass index (BMI) in COVID-19 patients have shown mixed results, including both the presence and absence of an association [10,11,12,13]. The literature examining this association in patients with neurodegenerative diseases is vast. In individuals with Parkinson’s disease, an association has been documented between severe chemosensory impairment in the late stages of disease and pronounced malnutrition [14]. Knopman et al. suggests that changes in BMI may be related to prodromal olfactory function deficits in women with dementia [15].

OD has the potential to negatively impact how individuals interact with food in many different ways. Fjaeldstad and Smith assessed individuals with quantitative and qualitative OD and found that these chemosensory disturbances are associated with decreased food-related quality of life, weight changes, decreased food recognition, decreased food liking, and even cooking difficulties [16]. Given that new-onset olfactory loss may affect anywhere from 18–43% of individuals with COVID-19, and that approximately 5% of these individuals experience persistent smell loss lasting at least 6 months after infection [17], additional research is needed to understand how these olfactory changes affect BMI, nutrition, and the chronic diseases that follow [18,19,20].

Retronasal olfaction, which is defined as the perception of smells that travel to the olfactory cleft via the nasopharynx during oral and oropharyngeal phases of food ingestion, constitutes much of what individuals perceive as flavor during food consumption [21]. The differential contributions of olfactory and gustatory loss after COVID-19 remain controversial; Hintschich et al. suggests that for the majority of individuals, the perception of gustatory dysfunction may be due to a misattribution of flavor perception to the sense of taste [22]. However, other evidence suggests that taste loss is a bona fide symptom of COVID-19 with a distinct mechanism [23]. In COVID-19, individuals report taste dysfunction when tested with stimuli that have few to no olfactory components [24]. Furthermore, mechanistic studies have demonstrated the presence of replicating SARS-CoV-2 in type II cells of taste buds in the oral cavity, which directly impacts taste receptor cells and affects taste perception [25]. This suggests that while taste and olfaction are affected by COVID-19 and both have impact on nutrition, the mechanisms of how the SARS-CoV-2 virus affects taste and olfactory receptors is different.

The purpose of this study is to examine how nutritional status changes over time through a longitudinal assessment of BMI in individuals with COVID-19-associated quantitative and qualitative OD. The researchers hypothesized that persistent OD would lead to long-term increases in BMI due to a greater dependence on factors to compensate for loss of flavor, such as amplified taste stimuli, spices, and food texture, along with the subsequent consumption of more calorie-dense foods. It is important to note that BMI is an imperfect measure as it indirectly measures body fat and has inherent racial bias [26,27].

## 2. Materials and Methods

### 2.1. Study Participants

Thirty-one study participants were recruited as part of an NIH-funded, prospective, longitudinal cohort study examining the relationship between COVID-19-associated olfactory changes and neurocognition. There were 13 males and 18 females with a mean age of 44.850 ± 15.426. Individuals were recruited primarily via otolaryngology clinic referral, with participants also volunteering after responding to flyers placed in the community and around Columbia University Irving Medical Center (CUIMC) and the online study participation platform RecruitMe hosted by CUIMC. Inclusion criteria were age 18 years or older, persistent subjective olfactory changes lasting beyond a three-month duration, and history of COVID-19 infection confirmed via PCR testing, serology, or clinical assessment if diagnosis was prior to widespread COVID-19 testing availability. Reasons for exclusion included pre-existing olfactory disturbance, chronic rhinosinusitis, failure to complete the online survey, sinonasal outcome test-22 (SNOT-22) score greater than 22 or any nasal domain question greater than five, olfactory cleft endoscopy scale greater than three, prior diagnoses of neurological disease or head trauma, moderate/moderately-severe depression assessed by PHQ-9, a diagnosis of diabetes or cancer, or food insecurity as assessed by the U.S. Household Food Security Survey Short Form [28,29]. All participant recruitment (protocol AAAT6202) was approved by an institutional review board through the CUIMC Human Research Protection Office.

### 2.2. Data Collection

Each participant completed two in-person evaluations one year apart. Data were collected between April of 2021 and March of 2023. All participants were first infected with SARS-CoV-2 up to the end of 2021. Participants underwent nasal endoscopy to rule out co-existent sinonasal disease in determining eligibility. Demographics, general medical history, COVID-19 history, food insecurity, Patient Health Questionnaire-9 (PHQ-9), and SNOT-22 assessments were collected via the online Research Electronic Data Capture (REDCap) data management system. Participants presenting with subjective OD were evaluated for the presence of quantitative OD and queried for the presence of qualitative OD. Participants underwent semi-objective psychophysical tests of olfaction using Sniffin’ Sticks (Burghart Messtechnik GmbH, Holm, Germany) [30]. Tests administered included threshold (T), discrimination (D), and identification (I) assessments, and individual scores were summed into a comprehensive TDI score. Based on prior data, participants can be classified as functional anosmics (TDI ≤ 16), hyposmics (TDI ≥ 16.25 and ≤30.5), normosmics (TDI ≥ 30.75 and ≤41.25), and supersmellers (TDI ≥ 41.5). Due to the limited population size in this study, quantitative OD encompassed all individuals with TDI ≤ 30.5, which includes hyposmics and anosmics, while those without OD were defined as TDI ≥ 30.75 to include normosmics and supersmellers [31]. Qualitative olfaction, or parosmia, was assessed utilizing a modified version of a questionnaire initially developed by Landis et al. [32]. If a participant answered “often” or “always” to “Because of my olfactory problem, food tastes different than it should taste” or “Odors which are pleasant to other people are unpleasant to me”, they were placed into the Parosmia cohort. At each study visit, participants’ heights and weights were measured. Any missing height and weight data were obtained via the electronic medical record from concomitant office visits.

Participant data were split into two groups in two independent ways: persistent versus transient OD, and parosmics versus non-parosmics. All participants met inclusion criteria of experiencing ≥ 3 months of subjective OD; however, persistent OD was defined via psychophysical assessment as a TDI score of 30.5 or less at two visits one year apart, and transient OD included all remaining subjects. The “Parosmia” subgroup includes all participants who reported qualitative smell dysfunction, and the “No parosmia” subgroup included those without coincident qualitative OD.

### 2.3. Statistical Analysis

Descriptive statistics were calculated as means and standard deviations for continuous variables. Within-subject changes in BMI were assessed using non-parametric Wilcoxon signed-rank tests. All statistical tests were two-sided with an *α* of 0.05. Statistical tests were carried out using SPSS software (IBM Corp. Released 2022. IBM SPSS Statistics for Windows, Version 29.0, Armonk, NY, USA: IBM Corporation). The Holm–Bonferroni method was implemented to adjust *α* levels for multiple comparisons.

## 3. Results

Thirty-one study participants were included in this analysis. This patient population reported primarily ambulatory COVID-19 infection, with only one participant reporting a previous hospitalization as a result of acute COVID-19. Overall participant characteristics in addition to characteristics grouped by quantitative or qualitative smell categorization can be found in Table 1.

**Figure 1:**
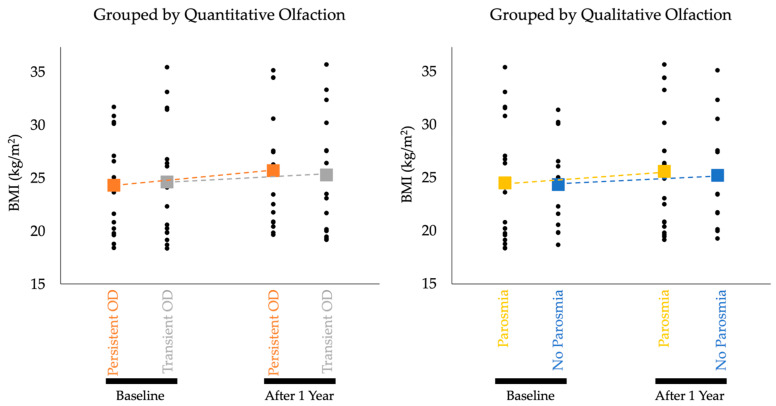
Aggregate BMI Data by Groups. Black dots are individual data points that represent individual participants’ BMI measurements. Dotted lines show the rate of change in average BMI in each cohort over one year. The change in BMI for both the Persistent OD and Parosmia cohorts achieved statistical significance.

Individual participant and aggregate mean BMI data by groups are documented in Figure 1. Study participants experiencing persistent quantitative OD (hyposmia or anosmia) over the course of one year (*n* = 15) showed a statistically significant increase in BMI from baseline to follow up (*p* = 0.004, adjusted *α* = 0.0125). Controls without persistent quantitative OD (*n* = 16) showed no statistically significant change in BMI over the same time period (*p* = 0.079, adjusted *α* = 0.05). Individuals with self-reported parosmia (“Parosmia”; *n* = 19) had a statistically significant increase (*p* = 0.011, adjusted *α* = 0.0167) in BMI over 1 year. Controls without self-reported parosmia (“No Parosmia”; *n* = 12) had no significant change in BMI (*p* = 0.028, adjusted *α* = 0.025). Findings are summarized in Table 2 and graphically depicted in Figure 1.

## 4. Discussion

This study recruited individuals with self-reported OD following COVID-19. Participants underwent medical evaluation and psychophysical olfactory testing at two time points. The results showed statistically significant increases in BMI after one year in individuals with quantitative hyposmia or anosmia or with self-reported parosmia. Statistical analysis showed no significant change in BMI in controls with transient olfactory loss or without self-reported parosmia.

This study further emphasizes the association between OD and increased BMI, and is one of the only clinical studies that specifically examines the relationship between COVID-19-associated OD and changes in BMI [9]. There is robust biochemical evidence supporting mechanisms of smell loss in obesity. Namely, there is dysregulation of ghrelin and leptin levels in obesity, both of which dampen central olfactory system function [33]. While this mechanism provides a basis for understanding OD in patients with longstanding obesity, there is likely a different mechanism accounting for disproportionate BMI increases in patients with post-COVID quantitative OD and parosmia. Specifically, the mechanism for underlying OD in obese patients may stem from metabolic dysregulation whereas the etiology of weight gain in patients with secondary OD likely arises from altered eating patterns in the setting of chemosensory dysfunction. A qualitative study of eating patterns among post-COVID patients highlighted a role for new-onset anosmia and parosmia in increased food intake to achieve flavor satisfaction while eating [34]. Additionally, multiple patients reported that they pursued “high-impact taste” to compensate for their sensory dysfunction—they cite foods high in sugar and salt as being more likely to achieve this outcome [34]. Thus, patients with OD must reach a higher threshold of flavor to achieve satisfactory taste. As such, these patients are more likely to develop a taste-focused diet, an eating pattern that is generally associated with unhealthier food choices [35]. This flavor threshold hypothesis provides a basis for understanding the disparate BMI changes between post-COVID patients with persistent OD or parosmia and those with only transient OD or a lack of parosmia.

The main outcome measure utilized in this study is BMI, which has certain limitations as a proxy for nutrition [26,27]. Benefits of using BMI as an outcome measure include availability of data, ease of calculation, and ability to quantify. Shortcomings of BMI include failure to (i) differentiate between muscle and fat, (ii) account for age-related changes, and (iii) account for sex-related variations [36]. And yet, a study by Moon et al. demonstrates that BMI measures negatively associate with fat intake in women and positively associate with intake of animal protein, suggesting that the result of increased BMI is consistent with the hypothesized dietary changes previously outlined [37]. Additionally, BMI has been shown to reflect nutritional quality of breakfast in children and adolescents, supporting the use of the measure as a proxy for nutrition [38].

The COVID-19 pandemic has altered lifestyle and eating habits of individuals [39,40]. During the beginning of the pandemic, guidance from government and health officials was to remain at home and isolate from others, leading to higher consumption of packaged foods in the setting of a more sedentary lifestyle [41]. Additionally, there were shortages of food with some individuals experiencing food insecurity [42]. A systematic review that looked at longitudinal changes in eating patterns throughout the pandemic found that eating habits were modified and characterized by increased snacking and a preference for more sweet and processed foods compared to before the pandemic [39]. The results from our study demonstrate that individuals with persistent COVID-19-associated OD, both in quantitative and qualitative realms, have significant increases in BMI as opposed to individuals without persistent OD. Some may argue that this increase in BMI is due to changes in lifestyle and eating habits that have occurred with the pandemic; however, the comparison groups include individuals who previously had a COVID-19 infection and would similarly be affected by the pandemic’s social impacts. Furthermore, studies documenting the effects of smell loss on eating behaviors have found that those who lose their sense of smell acutely have more dietary alterations than those who lose their sense of the smell gradually [43]. Velluzzi et al. found an inverse correlation between olfactory abilities and BMI and also showed that individuals with higher TDI scores were more likely to adhere to a Mediterranean diet (associated with lower BMI) [44]. Aschenbrenner et al. additionally found that in those with baseline OD, more individuals gain weight than lose weight [43]. Ferrulli et al. analyze several studies on body weight changes consequent to COVID-19-associated OD and found that weight changes occur bidirectionally with a slightly higher prevalence of weight loss versus weight gain [45]. Even though the statistically significant changes in BMI in our study were only in those with persistent quantitative OD and parosmia, all cohorts in our study gained weight, and the weight gain was most significant in the two cohorts with forms of OD. This global weight gain underscores the importance of the statistically significant weight gain among those with quantitative OD and parosmia.

The specific finding of a significant weight gain in the parosmia cohort warrants further discussion, as the flavor threshold hypothesis (i.e., eating more food to elicit a stronger flavor experience) for weight gain in a parosmia cohort is less intuitive given the general dissatisfaction these individuals have with common odors. While there is a sizable quantity of research examining the interaction between olfactory loss and weight changes, there are significantly fewer studies examining the impact of parosmia on weight changes. A recent study from Fjaeldstad and Smith (2022) investigated the presence and directionality of weight changes in individuals with olfactory changes stemming primarily from COVID-19 [16]. Individuals with a lower frequency of parosmia had greater weight *gain*. It is more intuitive that individuals experiencing parosmia (i.e., a “bad” or distorted perception of odors) could struggle to maintain a standard dietary intake if the pleasure they associate with food, an element strongly driven by olfactory ability, is diminished [46]. While the present study did not collect information on the frequency or severity of participants’ experiences with parosmia, it is possible that parosmia did not occur as frequently for these individuals in comparison to those in the Fjaeldstad and Smith (2022) study, given this study’s finding of a net weight gain in the setting of parosmia. Therefore, it could be important to understand the *frequency* of an individual’s experience with parosmia for clinicians to provide appropriate counseling on dietary strategies for this condition.

Individuals with poorer olfaction report experiencing a lower quality of life (QoL) than those with normal olfaction [47]. Moreover, up to one-third of patients with OD exhibit some form of depressive symptoms [47,48]. In this study, individuals with moderate or moderately-severe depression were excluded. Because changes in appetite are one of the hallmark symptoms of depression, this could be an important covariate to include in further analysis to delineate whether depressive symptoms are greatest in those with the largest changes in BMI. Understanding the psychological impacts of OD on individuals’ overall health and diet could provide another avenue for therapeutic intervention and allow clinicians to teach coping strategies to address psychological disturbances occurring secondary to chemosensory dysfunction.

Management of post-COVID-19 OD relies primarily on consistent olfactory training (OT), with several studies confirming the efficacy of OT for post-viral OD [49,50]. Given that an improvement in olfactory abilities into the normosmic range (i.e., the transient OD group in this study) appears to dampen the magnitude of BMI change, it is therefore important to support patients in adhering to olfactory training in an effort to restore the sense of smell and alleviate the effects of OD on BMI. In addition to advocating for sustained OT, clinicians should focus on counseling patients to prioritize non-chemosensory food attributes (i.e., texture and mouthfeel) to enhance dietary satisfaction in the setting of persistent OD [51].

One of the advantages of this study involves the use of the extended Sniffin’ Sticks test to assess olfactory function. Unlike more abbreviated smell assessments, which often only assess odor identification abilities, the extended Sniffin’ Sticks test is a psychophysical assessment that allows for a more nuanced understanding of individuals’ olfaction and allows for the classification of olfactory status based on the composite TDI score rather than relying on any one domain. Another advantage of this study is the length of time between study visits for the assessment of olfaction and BMI. Much of the current literature focuses on the association between OD and BMI within a shorter time span than that included in the present study [45], further highlighting the importance of longitudinal follow-up for individuals with persistent COVID-19-associated OD or parosmia [45].

This study is not without limitations: sample size and statistical methods employed restrict the definitive conclusions that can be drawn from this work, including direct group comparisons and cause-and-effect claims. Additionally, the racial and ethnic diversity of this study population may not reflect the broader population of those individuals globally suffering from COVID-19-associated OD. This study involved a degree of recruitment bias, given that many patients were recruited after seeking clinical care for OD in an outpatient ENT practice. Therefore, the proportion of individuals with OD and/or parosmia is overrepresented compared to a random sample [52,53]. We are also limited in drawing strong conclusions regarding whether accelerated weight gain occurs only during times of smell loss or also after smell has normalized, given that we collect participant BMI measurements at two time points. We do not have temporal data regarding the rates at which patients transitioned from parosmia to no parosmia, as patients were classified in these groups depending on baseline characteristics. While it is logical that several of the parosmia patients may have also experienced persistent OD, there is nonetheless a probable independent contribution of parosmia to weight gain given that the parosmia and no parosmia cohorts normalize to what is functionally the same TDI score. Finally, it is difficult to isolate the effect of OD on BMI; however, this study utilizes control cohorts with similar characteristics to mitigate confounding. Future research should seek to address these shortcomings and investigate further the specific dietary and lifestyle alterations that accompany nutritional changes in individuals with olfactory disorders.

## 5. Conclusions

Individuals with persistent OD after COVID-19, both quantitative and qualitative, experienced significant increases in BMI over the course of one year. Controls that lacked these olfactory changes did not have significant alterations in BMI during the same time period. Thus, OD may play a role in altering nutrition and dietary habits in this patient population. Counseling patients with olfactory changes after COVID-19 may be beneficial in mitigating long-term consequences of OD; however, larger studies are needed to further evaluate the relationship between OD and BMI.

## Figures and Tables

**Table 1:** Demographics.

Characteristic	All (*n* = 31)	Quantitative Smell		Qualitative Smell	
		Persistent OD (*n* = 15)	Transient OD, Controls (*n* = 16)	Parosmia (*n* = 19)	No Parosmia, Controls (*n* = 12)
Age at Baseline (mean ± SD)	44.850 ± 15.426	45.410 ± 16.251	44.324 ± 15.127	43.501 ± 13.724	46.985 ± 18.240
*Sex Assigned at Birth*					
Female	18	6	12	12	6
Male	13	9	4	7	6
*Race*					
Asian	3	1	2	1	2
Black/African American	2	1	1	1	1
White	22	12	10	13	9
Unknown/ Declined to Answer	4	1	3	4	0
*Ethnicity*					
Hispanic/Latino	4	1	3	4	0
Non-Hispanic/Latino	27	14	13	15	12
*Perceived COVID-19 Severity*					
Substantially Less Severe	12	6	6	6	6
Slightly Less Severe	8	3	5	4	4
Same as Others	5	1	4	4	1
Slightly More Severe	3	3	0	3	0
Substantially More Severe	1	1	0	1	0
Unknown/Not Reported	2	1	1	1	1

**Table 2:** Change in BMI After One Year for Participants with and Without Persistent OD and Parosmia.

	Olfaction at Baseline (Mean ± SD)	Olfaction after 1 Year (Mean ± SD)	ΔBMI (kg/m^2^)	Test Statistic (Z)	Adjusted α	*p*-Value
*Persistent OD* (*n* = 15)						
TDI Score	22.917 ± 5.948	24.400 ± 5.162	1.377	−2.897	0.0125	0.004 *
Threshold	4.050 ± 2.527	6.200 ± 2.910				
Discrimination	10.067 ± 2.658	9.733 ± 2.542				
Identification	8.800 ± 2.274	8.467 ± 2.029				
*Transient OD*, controls (*n* = 16)						
TDI Score	29.875 ± 5.350	32.828 ± 3.625	0.650	−1.758	0.05	0.079
Threshold	6.625 ± 2.676	9.891 ± 2.515				
Discrimination	12.188 ± 2.482	12.063 ± 2.144				
Identification	11.063 ± 2.516	10.875 ± 2.604				
*Parosmia* (*n* = 19)						
TDI Score	25.421 ± 6.533	28.789 ± 4.534	1.082	−2.535	0.0167	0.011 *
Threshold	5.263 ± 2.933	7.789 ± 3.428				
Discrimination	10.947 ± 2.592	11.421 ± 2.317				
Identification	9.211 ± 2.573	9.579 ± 1.953				
*No parosmia*, controls (*n* = 12)						
TDI Score	28.229 ± 6.560	28.688 ± 8.403	0.876	−2.197	0.025	0.028
Threshold	5.563 ± 2.902	8.604 ± 3.176				
Discrimination	11.500 ± 3.060	10.167 ± 3.010				
Identification	11.167 ± 2.329	9.917 ± 3.554				

* Change is statistically significant (*p* < *α*).

## Data Availability

The data presented in this study are available on request from the corresponding author.
